# *In Vitro* Activity of a Novel Glycopolymer against Biofilms of Burkholderia cepacia Complex Cystic Fibrosis Clinical Isolates

**DOI:** 10.1128/AAC.00498-19

**Published:** 2019-05-23

**Authors:** Vidya P. Narayanaswamy, Andrew P. Duncan, John J. LiPuma, William P. Wiesmann, Shenda M. Baker, Stacy M. Townsend

**Affiliations:** a Synedgen, Inc., Claremont, California, USA; b Willamette University, Salem, Oregon, USA; c University of Michigan, Department of Pediatrics and Communicable Diseases, Ann Arbor, Michigan, USA; d Townsend Bio-Pharm Consulting, Rancho Cucamonga, California, USA

**Keywords:** *Burkholderia*, PAAG, antimicrobial activity, antimicrobial agents, biofilms, confocal microscopy, glycopolymer

## Abstract

Burkholderia cepacia complex (Bcc) lung infections in cystic fibrosis (CF) patients are often associated with a steady decline in lung function and death. The formation of biofilms and inherent multidrug resistance are virulence factors associated with Bcc infection and contribute to increased risk of mortality in CF patients. New therapeutic strategies targeting bacterial biofilms are anticipated to enhance antibiotic penetration and facilitate resolution of infection. Poly (acetyl, arginyl) glucosamine (PAAG) is a cationic glycopolymer therapeutic being developed to directly target biofilm integrity. In this study, 13 isolates from 7 species were examined, including Burkholderia multivorans, Burkholderia cenocepacia, Burkholderia gladioli, Burkholderia dolosa, Burkholderia vietnamiensis, and B. cepacia. These isolates were selected for their resistance to standard clinical antibiotics and their ability to form biofilms *in vitro*. Biofilm biomass was quantitated using static tissue culture plate (TCP) biofilm methods and a minimum biofilm eradication concentration (MBEC) assay. Confocal laser scanning microscopy (CLSM) visualized biofilm removal by PAAG during treatment. Both TCP and MBEC methods demonstrated a significant dose-dependent relationship with regard to biofilm removal by 50 to 200 μg/ml PAAG following a 1-h treatment (*P* < 0.01). A significant reduction in biofilm thickness was observed following a 10-min treatment of Bcc biofilms with PAAG compared to that with vehicle control (*P* < 0.001) in TCP, MBEC, and CLSM analyses. PAAG also rapidly permeabilizes bacteria within the first 10 min of treatment. Glycopolymers, such as PAAG, are a new class of large-molecule therapeutics that support the treatment of recalcitrant Bcc biofilm.

## Abstract (precis)

Burkholderia cepacia complex (Bcc) lung infections in cystic fibrosis (CF) patients are often associated with a steady decline in lung function and death. The formation of biofilms and inherent multidrug resistance are virulence factors associated with Bcc infection and contribute to increased risk of mortality in CF patients.

## INTRODUCTION

The Centers for Disease Control and Prevention ranked chronic respiratory disease as one of the leading causes of death (300,000/year) in the United States in 2017. Accumulation of viscous pulmonary mucus reduces mucociliary clearance in diseases such as chronic obstructive pulmonary disease (COPD) and cystic fibrosis (CF). CF is the most common inherited disease among Caucasians and results from one of many types of defects of the cystic fibrosis transmembrane conductance regulator (CFTR) ion channel. Reduced mucociliary clearance in the CF lung is manifested by frequent infections and chronic inflammation leading to irreversible lung damage (1).

Burkholderia cepacia complex (Bcc) is a subgroup within the *Burkholderia* genus consisting of 20 species and is known to be opportunistic and causative of severe lower respiratory infections in patients with CF (1–5). Recurrent or chronic Bcc infections result in a decline of pulmonary function, frequent exacerbations, and high rates of mortality. Bcc-infected patients are often excluded from the option of lung transplantation (6–12). *Burkholderia* species grow in the mucus of the CF lung, develop biofilms, (6, 12–14) and often appear later in the progression of CF (15). Viscous pulmonary mucus and thick biofilms impede antibiotic penetration in the CF airway and reduce antibiotic treatment efficacy. Though breakthrough treatments have added years to the lives of people with cystic fibrosis, increased incidence of Bcc infection, bronchiectasis, and pulmonary hypertension have emerged as a consequence of longevity (16). The development of new approaches that target biofilm infections, including large molecule glycopolymer therapies, offers a unique opportunity to extend the life expectancy of CF patients even further.

The treatment of Bcc pulmonary infection is severely limited by inherent antibiotic resistance, augmented by the propensity for biofilm development that protects Bcc from immune clearance and contributes to the persistence of infection in the CF airway (17). Clinical isolates of Bcc demonstrate intrinsic resistance to aminoglycoside antibiotics and high levels of beta-lactams due to inducible chromosomal beta-lactamases and altered penicillin-binding proteins (18). These innate bacterial virulence factors exacerbate recalcitrance to therapeutic antibiotics and, in combination with ineffective CF host defenses, result in severe and frequently fatal chronic lung infections (17, 19). Bcc infections associated with CF are typically treated with a combination of antibiotics that often include tobramycin, meropenem, ceftazidime, piperacillin, cefepime minocycline, tigecycline, or trimethoprim-sulfamethoxazole. However, with little clinical data available to support specific treatment regimens, clinicians often rely on *in vitro* data and case reports to support novel therapies with limited success (20, 21).

The increasing incidence of multidrug resistant infections requires novel drug development and treatment strategies to facilitate treatment of infection without supporting the development of antibiotic resistance. Poly (acetyl, arginyl) glucosamine (PAAG) is among a recently discovered novel class of glycopolymer therapeutics that demonstrates antibacterial activity, permeabilizes bacterial membranes, and potentiates antibiotics against drug-resistant bacteria (22–24). Glycopolymers are typically nontoxic and have been developed for wound dressing technology, hemostatic therapeutics, and drug delivery without significant risk (25–29). Divalent cations are known to support the structure of the outer membrane of Gram-negative bacteria, preventing charged antibiotic molecules from crossing the membrane. PAAG, like other cationic antimicrobials, is hypothesized to outcompete these divalent cations for binding to the bacterial outer membrane (30). The antibacterial activity of PAAG against Gram-negative bacteria was enhanced when PAAG was more densely charged, facilitating rapid depolarization of bacterial membranes (23). Biofilm disruption, enhanced antibiotic activity, and increased mucociliary clearance following PAAG treatment were recently demonstrated and support PAAG as a representative in a novel class of glycopolymers capable of disrupting biofilms. Additionally, PAAG has been found to demonstrate significant disruption of static biofilms in tissue culture plate (TCP) assays with nontuberculous mycobacteria (NTM) (data not shown). Potentiation of antibiotic activity and sensitization of drug-resistant Bcc to tobramycin and meropenem indicate that inhaled glycopolymer therapeutics, such as PAAG, could facilitate antibiotic penetration (24). This study addresses the ability of PAAG to penetrate and rapidly reduce cohesion in biofilms of Bcc and to permeabilize the bacteria associated with Bcc biofilms.

The persistence of Bcc biofilms in the lungs of CF patients limits treatment options and highlights the need for antibiofilm therapeutic strategies. In this study, the antibiofilm activity of PAAG was assessed against biofilms produced by a number of clinically relevant Bcc isolates from CF patients. The ability of PAAG to dissipate preformed Bcc biofilms was determined by tissue culture plate (TCP) and minimum biofilm eradication concentration (MBEC) assays and visually confirmed using confocal laser scanning microscopy (CLSM). Fluorescently labeled PAAG was used to characterize its diffusion in the biofilms and spatial relationship to viable bacteria.

## RESULTS

### Characterization of antimicrobial activity against planktonic bacteria.

A genetically and geographically diverse collection of Bcc isolates was obtained from the Burkholderia cepacia Research Laboratory and Repository (BcRLR). Isolates from both males and females of various ages were represented in this collection. An MIC assay was completed to characterize the level of antibacterial resistance among the strains tested in this study. Three commonly used therapeutic antibiotics, meropenem, tobramycin, and ceftazidime, were evaluated. A majority of the Bcc isolates were resistant to the antibiotics tested according to CLSI guidelines (Table 1) (31). Meropenem resistance was observed in 69% (*n* = 8) of isolates tested and showed no obvious pattern with respect to species or patient background. Tobramycin resistance was observed in 69% (*n* = 9) of isolates tested. Interestingly, all of the Burkholderia gladioli isolates tested were sensitive to tobramycin. Ceftazidime resistance was observed in 53% (*n* = 7) of isolates tested and, again, all of the *B. gladioli* isolates were sensitive to ceftazidime. Burkholderia vietnamiensis was sensitive to all three antibiotics. B. cepacia was resistant to all three antibiotics.

**TABLE 1:** Antimicrobial susceptibility of the Bcc clinical isolates tested

*Burkholderia* species (no. of isolates)	% resistant to:^*a*^		
	MEM	TOB	CAZ
B. cenocepacia (3)	67	100	67
*B. gladioli* (3)	100	0	0
*B. multivorans* (4)	75	100	75
*B. vietnamiensis* (1)	0	0	0
*B. dolosa* (1)	0	100	100
B. cepacia (1)	100	100	100
Total (13)	69	69	53

a The respective resistance breakpoints were as follows: meropenem (MEM) **>** 16 μg/ml, tobramycin (TOB) **>** 16 μg/ml, ceftazidime (CAZ) **>** 32 μg/ml.

### Effects of PAAG on preformed biofilms.

**FIG 1:**
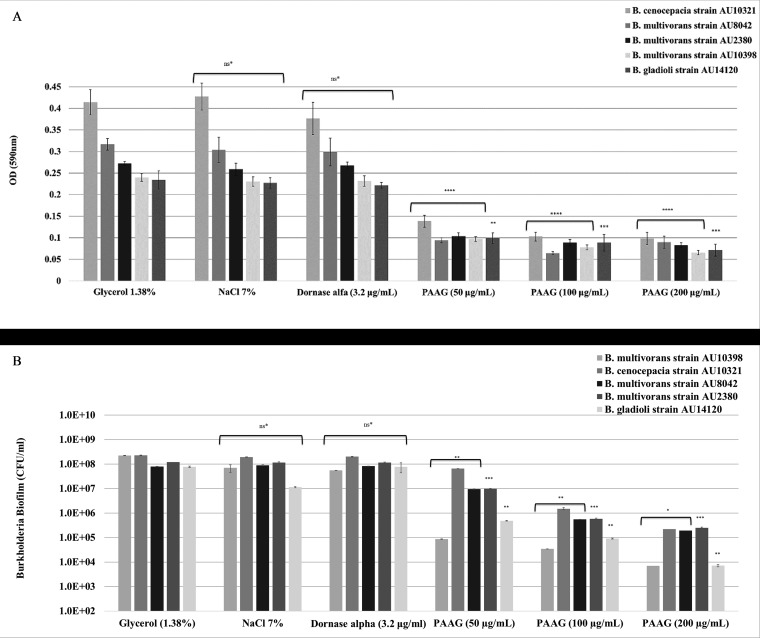
Biofilm removal of Bcc clinical isolates. Comparative *Burkholderia* biofilm removal data after treatment with PAAG for 1 h. The biofilm density was quantitated using crystal violet staining in a static (TCP) biofilm assay (A). Data shown are the mean optical density (OD) ± the standard error (SE). The biofilms were also quantitated by viable counts using the MBEC assay (B). Data shown are the mean CFU/ml ± SE. **, *P* < 0.01; ***, *P* < 0.001; ****, *P* < 0.0001. ns*, data not significantly different from the vehicle control (*P* > 0.05).

The ability of PAAG to disrupt biofilms was tested using the TCP assay and MBEC analysis of mature Bcc biofilms. Static Bcc biofilms (grown for 48 h) were treated with PAAG (50 to 200 μg/ml), hypertonic saline (7%), or dornase alfa (3.2 μg/ml) for 1 h. The biofilms were rinsed with sterile water, stained with 1% crystal violet, and the optical density was read at 590 nm. Quantification of the remaining biofilm biomass after rinsing showed a significant dosage effect in the removal of preformed biofilms following PAAG treatment and no significant reduction following treatment with hypertonic saline or dornase alpha (Fig. 1A). Treatment with PAAG resulted in significant reductions in Bcc biofilm biomass (*P* < 0.001) in a dose-dependent manner. However, variability was observed in the effect of PAAG on the biofilms of different isolates. The effect of PAAG on Burkholderia cenocepacia showed the most significant biofilm reduction (*P* < 0.001). Both hypertonic saline and dornase alpha had no significant influence on preformed biofilm removal compared to that of the vehicle control.

In addition to measurement of biofilm mass, the number of remaining viable bacteria was assessed using the MBEC assay (MBEC physiology & genetics [P&G] assay; Innovotech). These studies complement the quantification of preformed biofilm biomass removal by measuring the CFU per milliliter that are present after biofilm disruption and rinsing. A significant trend in dose-dependent reduction in bacterial viability was achieved with a 2- to 3-log reduction in CFU per milliliter following a 1-h treatment (Fig. 1B). Variability was observed in the magnitude of PAAG’s ability to disrupt biofilms of different strains of *Burkholderia*, although all biofilms are significantly reduced relative to control. The MBEC assay shows that glycerol control (1.38%), hypertonic saline (NaCl 7%), and dornase alpha (3.2μg/ml) have no effect on the amount of remaining bacteria after rinsing.

### PAAG activity against preformed biofilms observed by CLSM.

**FIG 2:**
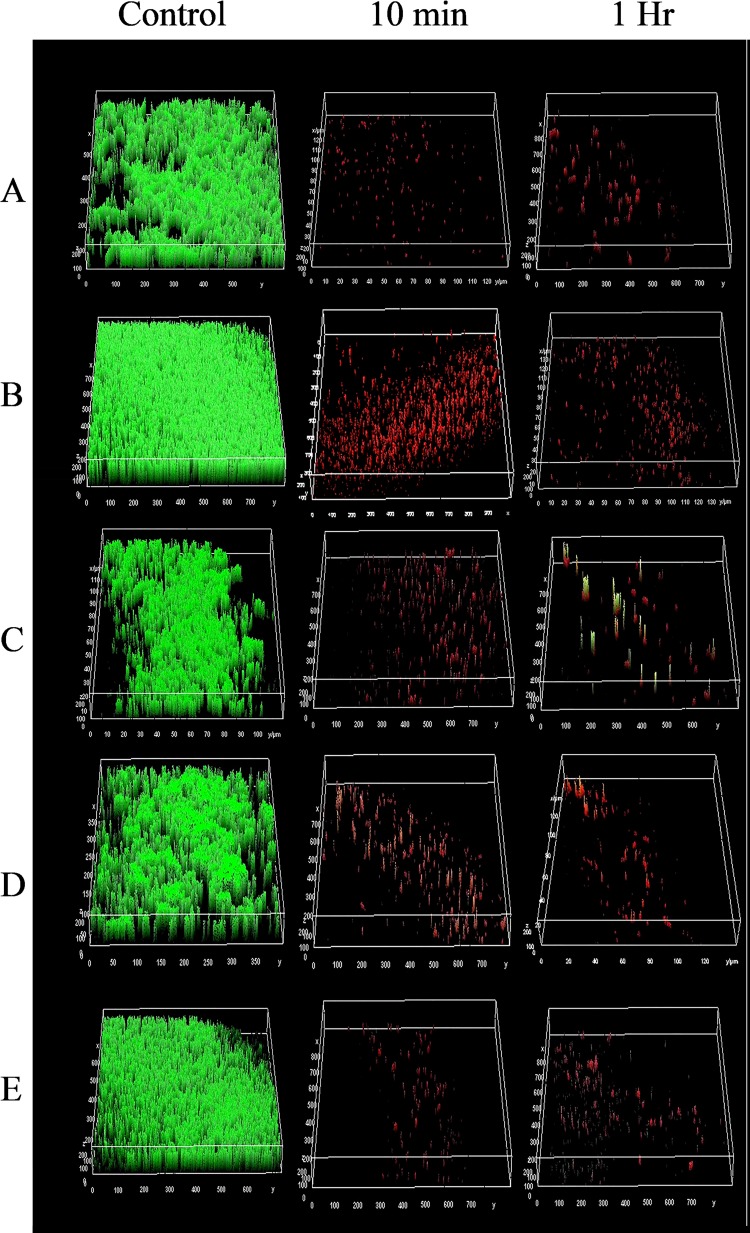
Confocal images of PAAG-treated Bcc biofilms. Representative images of LIVE/DEAD stained Bcc biofilms treated with vehicle control or 200 μg/ml of PAAG for 10 min or 1 h visualized by CLSM. *B. gladioli* strain AU10529 (A), B. cenocepacia strain 10321 (B), biofilms of *B. dolosa* strain BC35 (C), *B. multivorans* strain 10398 (D), and B. cepacia ATCC 25416 (E) (green, live; red, dead). Scale bar = 10 μm. The average thickness measurement for the control biofilms was 39.2 μm.

**FIG 3:**
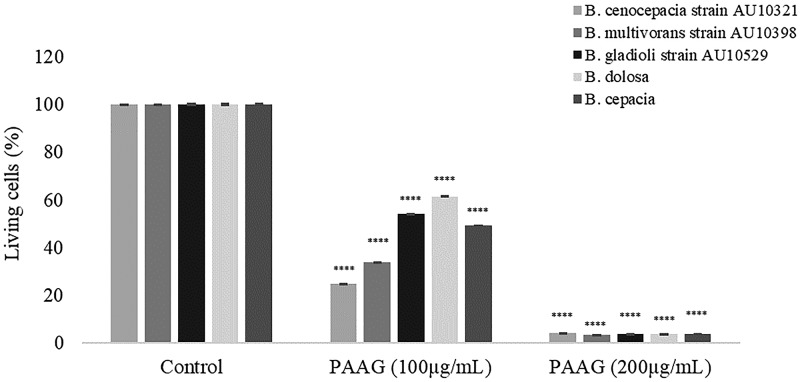
Quantification of live bacteria in PAAG-treated Bcc biofilms. CLSM was used to measure the number of LIVE labeled (SYTO 9 labeled) bacteria following a 1-h treatment with PAAG or vehicle control (1.38% glycerol). ****, *P* < 0.0001.

Mature Bcc biofilms were formed on glass coverslips and exposed to vehicle control (1.38% glycerol, pH 7.4) and PAAG at 200 μg/ml. The biofilms were rinsed by gently submerging the coverslips at a 90° angle in water for 1 s. The remaining biofilms were observed by CLSM after staining with SYTO 9/propidium iodide (LIVE/DEAD) fluorophores. Figure 2 shows three-dimensional images of 5 representative Bcc biofilms treated with PAAG for 10 min or 1 h compared to those of the control-treated biofilms. Quantitative analysis of the percentage of living cells (SYTO 9 labeled) is shown in Fig. 3. A dose-dependent reduction in biofilm mass was observed upon treatment with PAAG. A 15- to 20-fold reduction or 25-fold reduction in living cells was observed following a 10-min or 1-h treatment with 200 μg/ml PAAG, respectively, for all the strains tested (Fig. 3). Significant reductions in living cells were observed at lower concentrations of PAAG (200 μg/ml) even with a significantly mature biofilm grown for 48 h (data not shown). PAAG permeabilized bacteria as demonstrated by the change in cell color from green to red and significantly reduced the number of living cells in both the 10-min and 1-h treatments.

### Effect of PAAG on biofilm thickness.

**FIG 4:**
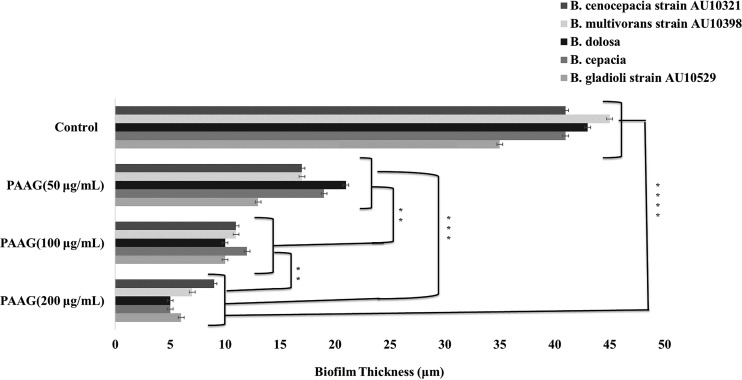
Bcc biofilm thickness reduction. Light microscopy was used to measure the optical biofilm thickness. The biofilms were treated with PAAG for 1 h and then the biofilm thickness was translated into physical thickness based on biofilm refraction measurements. **, *P* < 0.01; ***, *P* < 0.001; ****, *P* < 0.0001.

The biofilm thicknesses of the vehicle control- and PAAG-treated biofilms were optically measured and assessed assuming the refractive index (RI) of the biofilm was equal to water per Bakke and Olsson (32). Five replicates (*n*) were used for each data point. Figure 4 shows that PAAG significantly decreased biofilm thickness from approximately 40 μm to a range of 5 to 10 μm within 1 h of treatment. Significant reductions in biofilm thickness were observed on mature biofilms grown for 48 h at the lower PAAG concentrations (100 to 200 μg/ml) for all *Burkholderia* species tested. Exposure to PAAG at 200 μg/ml resulted in a significant decrease (*P* < 0.001) in biofilm thickness compared to that of the 50 μg/ml PAAG treatment (*P* < 0.01) in a dose-dependent manner. An 80% reduction in biomass was observed that confirmed data obtained with the MBEC (CFU/ml) and TCP (biomass) assays.

**FIG 5:**
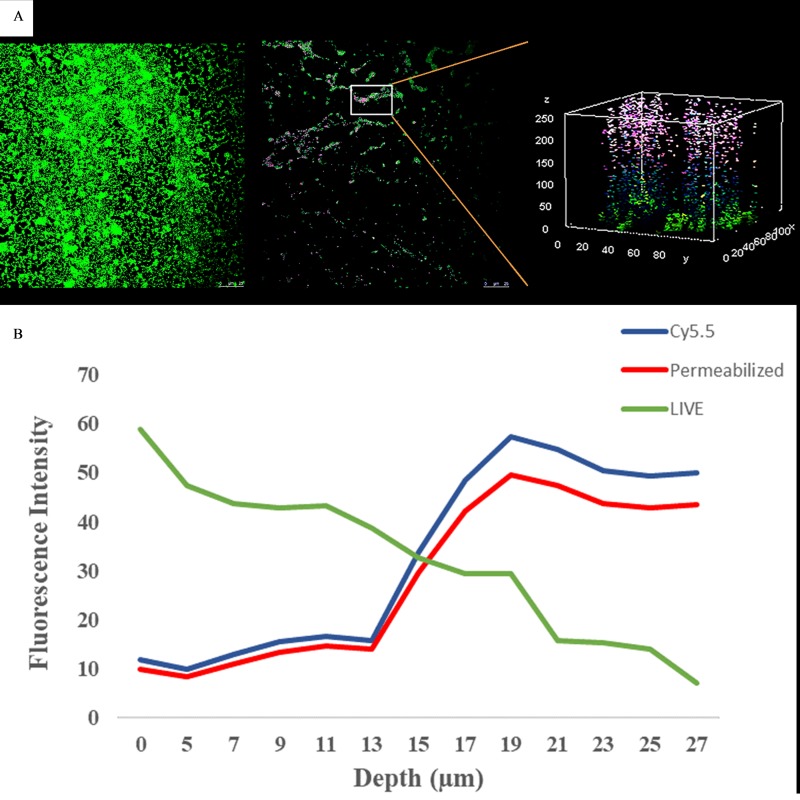
Confocal image and fluorescent quantification of Cy5.5-labeled PAAG. Representative images of LIVE/DEAD stained B. cenocepacia AU10321 biofilms treated with 200 μg/ml of PAAG-Cy5.5 for 1 h and visualized by CLSM. (A) B. cenocepacia strain 10321 biofilm treated with vehicle control (1.38% glycerol) (left). B. cenocepacia biofilm treated with 200 μg/ml of PAAG-Cy5.5 for 1-h (middle). Three dimensional CSLM image of the PAAG-Cy5.5-treated and stained biofilm (right) showing colocalization of Cy5.5 and propidium iodide on the surface of the biofilm compared to that of SYTO 9-labeled cells. All images show B. cenocepacia stained with SYTO 9/propidium iodide and visualized by CLSM. (B) Quantification of the fluorescent intensity of PAAG-Cy5.5 (blue line), permeabilized bacteria (red line), and live cells (green line) with respect to B. cenocepacia biofilm depth. Scale bar = 25 μm.

Mature B. cenocepacia AU10321 biofilms (48-h growth) were formed on glass coverslips and exposed to PAAG-Cyanine5.5 (Cy5.5) conjugate for 1 h along with vehicle-treated biofilms as a control. The biofilms were observed with a confocal microscope after staining with SYTO 9/propidium iodide (LIVE/DEAD) fluorophores. The confocal images were processed with ImageJ software (33). The three-dimensional image was selected from an area on the surface where some treated biofilm remained. Quantitative analysis of the fluorescence intensity (*y* axis) of living bacterial cells (SYTO 9 labeled) and permeabilized cells (propidium iodide labeled) and PAAG-Cy5.5 relative to the distance from the surface (*x* axis) is shown in Fig. 5B. In Fig. 5A, a small piece of remaining biofilm was studied in more detail to demonstrate the penetration of PAAG. The number of permeabilized bacteria increased as the distance from the surface decreased, demonstrating that living bacteria were more frequently observed deeper within the biofilm. The number of permeabilized bacteria increased as the distance from the surface increased, demonstrating that permeabilized bacteria were more frequently observed near the surface of the biofilm. The PAAG-Cy5.5 conjugate decreased as the distance from the surface increased and colocalized with the permeabilized fluorescent intensity marker propidium iodide, indicating that permeabilized cells were collocated with PAAG.

## DISCUSSION

These studies focus on a small but highly lethal genus of bacteria that is a growing concern to the CF community. Six representative species from among the Bcc were used to characterize the ability of PAAG to disrupt biofilms of very different *Burkholderia* strains. While not exhaustive, these strains represent a diverse set of frequently isolated species associated with CF patient lung infections. The chronic nature of CF lung infections contributes to a significant variability in bacterial phenotypes, such as the development of mucoid phenotype and biofilm exopolysaccharide (EPS) composition (34). Current antibiotic strategies for Bcc treatment focus on administration of antibiotic combination therapy and are intended to eliminate the bacteria from the lung. However, the use of antibiotics in CF has led to the chronic suppression of bacterial opportunists in the airway without their eradication and often leads to the development of antibiotic resistance (35). Heterogeneous populations of pathogenic bacteria, such as Bcc, add another layer of complexity when evaluating effective therapeutic options and the specificity of disease diagnosis. Identification of broadly effective therapeutics that target biofilms is needed to facilitate effective antibiotic usage and biofilm clearance.

This study characterized the ability of PAAG to disrupt the structure of preformed biofilms to allow for clearance or removal as a novel therapeutic strategy against Bcc lung infections. Biofilm disruption was independently measured by biomass, viable bacteria count, and visualization. PAAG was able to significantly facilitate disruption and removal of preformed biofilms from all six *Burkholderia* clinical species frequently associated with CF lung infections (Fig. 1 and 2). Mucolytic treatments (hypertonic saline and dornase alpha) were not able to disrupt mature Bcc biofilms (Fig. 1 and 2). PAAG targets the polymeric components of biofilms, differentiating it from standard mucolytic treatments that are not designed to interact with biofilms. The treated Bcc biofilms and vehicle controls were also visualized through CLSM, independently validating PAAG antibiofilm activity and showing that biofilm disruption and removal occurred within 10 min of treatment (Fig. 2). While the TCP and MBEC biofilm assays demonstrated a reduction in biomass and CFU per milliliter, LIVE/DEAD studies validated this observation by showing a 25-fold reduction in viable Bcc cells (Fig. 3) that was associated with an 80% reduction in biofilm thickness (Fig. 4). Imaging with LIVE/DEAD fluorescent dyes showed rapid disruption and subsequent removal of Bcc biofilms following a 10-min PAAG treatment and suggests that effective therapeutic treatment time may be short (Fig. 2). However, these *in vitro* analyses may not fairly represent the complexities of the CF lung and dynamics that exists between the lung microbiota, pathogens, and host factors. Active treatment in the lung is needed to translate these dramatic *in vitro* observations to potential outcomes in the clinic.

The protection provided by Bcc biofilms is a key virulence factor that contributes to impaired immune clearance and therapeutic treatment failures and facilitates chronic infection and inflammation that contributes to CF lung pathology. Bcc biofilms grow in the thick mucus of the CF lung, further complicating antibiotic access. PAAG may facilitate biofilm disruption by reducing biofilm EPS cohesion, but there are no published data on the mechanism of this effect. Although the current study did not include evaluating PAAG antibiofilm activity in the presence of mucus, PAAG is hypothesized to reduce mucus viscosity and to improve mucociliary clearance (36). Disrupting the interactions of the EPS molecules within biofilms may serve as a more efficient therapeutic strategy than targeting the interaction of the bacteria with the EPS (37).

In addition to biofilm-disrupting capability, the ability of PAAG to permeabilize Bcc was also reproducible among the six different species tested. The ability of PAAG to permeabilize Bcc bacteria within biofilms was confirmed by the observed colocalization of labeled PAAG with permeabilized bacteria, as highlighted by the LIVE/DEAD stain (Fig. 5). The outer membrane (OM) of Bcc significantly reduces the penetration of antibiotics into the cell, a function of Bcc’s innate antibacterial resistance (34). The barrier is comprised of polyanionic lipopolysaccharides (LPS) that require stabilizing divalent cations (Mg^2+^ or Ca^2+^) to maintain the cell membrane integrity. Previous work demonstrates PAAG’s ability to rapidly permeabilize the Escherichia coli membrane (23). Cationic antibiotics, such as polymyxin, displace magnesium ions associated with the LPS, disrupting the membrane and causing it to leak. The use of polycationic peptides to facilitate potentiation of antibiotics is a known strategy but is limited in practice by the toxicity of polycationic antimicrobial peptides (38). PAAG appears to be effective in permeabilizing bacteria and demonstrated limited cytotoxicity compared to that of the antimicrobial peptides, perhaps due to the fundamentally different structural features of a polyglucosamine rather than polypeptide backbone (23, 38, 39). The molecular disruption of the OM by polycationic PAAG through ion displacement is a physical interaction that facilitates bacterial permeabilization and supports antibiotic synergy by allowing antibiotic penetration or by reducing the bacteria’s fitness (34, 40). PAAG is anticipated to potentiate the activity of antibiotics against planktonic Bcc and not interfere with standard of care treatment. This study shows that PAAG has a broad activity to permeabilize all tested Bcc species in biofilms; however, further mechanistic studies are ongoing to understand the breadth of PAAG interactions with a more diverse set of bacteria.

The ability of PAAG to overcome a key clinical challenge in treating Bcc lung infections could provide significant gains in supporting positive clinical outcomes and preserving the quality of life of CF patients with chronic Bcc infection. The rapid disruption of mature Bcc biofilm cohesion by PAAG supports its use in combination with standard-of-care antibiotics during treatment of identified infection or exacerbations (41). Current antibiotic strategies guided by susceptibility testing can have significant side effects, still be subject to or facilitate the development of antibiotic resistance, and fail to support restoration of baseline lung function following exacerbation in 25% of patients (42). Narrowly focused antibiotic treatments may not fully consider the pathological impact of the biofilm in CF lung infections. The development of PAAG for clinical use has the potential to significantly improve the treatment of recalcitrant Bcc biofilm infections associated with CF and to potentiate the activity of antibiotics. Further investigations are ongoing to elucidate PAAG’s potentiation effects with biofilms and its clinical significance during CF-associated lung infections and exacerbations.

## MATERIALS AND METHODS

### Bacterial strains.

Thirteen Bcc strains were used in this study and are shown in Table 2. Eleven CF-relevant *Burkholderia* isolates were acquired from the Cystic Fibrosis Foundation (CFF) (BcRLR). These included *Burkholderia multivorans* (*n* = 4), B. cenocepacia (*n* = 3), *B. gladioli* (*n* = 3), and *B. vietnamiensis* (*n* = 1) and were commonly associated with infection in CF patients. These strains were recovered from eleven CF patients in the United States between 1995 and 2007. One deidentified *Burkholderia dolosa* (*n* = 1) CF isolate was acquired from the CF Isolate Core at Seattle Children’s Research Institute (J. Burns), and one environmental isolate, B. cepacia (*n* = 1), was obtained from ATCC.

**TABLE 2:** Cystic fibrosis clinical isolates used in this study

Species	Strain	Age (yrs) of patient	Patient sex^*a*^	Yr isolated	State
B. cenocepacia	AU0007	18	F	1995	NY
B. cenocepacia	AU0037	21	M	1996	PA
B. cenocepacia	AU10321	34	F	2005	WA
*B. gladioli*	AU10529	13	F	2005	OH
*B. gladioli*	AU14114	19	M	2007	OH
*B. gladioli*	AU14120	13	M	2007	FL
*B. multivorans*	AU0064	16	M	1997	OH
*B. multivorans*	AU2380	24	F	2000	OK
*B. multivorans*	AU8042	18	F	2004	WA
*B. multivorans*	AU10398	33	M	2005	WA
*B. vietnamiensis*	AU3251	15	F	2001	CA
*B. dolosa*	BC35				WA
B. cepacia	ATCC 25416				WA

a F, female; M, male.

### Bacterial culture conditions.

The bacteria were grown in Mueller-Hinton broth (MHB) and stored at −80° with 15% glycerol and recovered from the frozen stock following overnight incubation at 37°C on Mueller-Hinton (MH) agar (Difco). Viable plate counts used to quantify CFU were also grown on MH agar (Difco). Sterile water was used to briefly wash the biofilms. Pharmaceutical grade (USP) vehicle control solutions, glycerol (Spectrum), NaCl (Sigma), and dornase alpha (Sigma), were used. The treatment concentrations of the hypertonic saline (7%) and dornase alpha (3.2 μg/ml) used were biologically relevant (43).

### PAAG glycopolymer.

The polycationic proprietary glycopolymer is a polycationic arginine derivative of a natural polysaccharide poly-*N*-acetylglucosamine (PAAG) soluble at physiologic pH and is being developed as SYGN113 by Synedgen, Inc. (Claremont, CA). To label PAAG, Cyanine5.5 (Cy5.5) *N*-hydroxysuccinimide (NHS) ester (Lumiprobe, Hollandale Beach, FL) was conjugated to PAAG by mixing a 100:1 ratio of PAAG/Cy5.5 (by mass) overnight at room temperature, protected from light. The solution was dialyzed against sterile water and lyophilized.

### Antimicrobial susceptibility testing.

The antimicrobial agents used (meropenem, tobramycin, and ceftazidime) were all USP grade and obtained from Sigma-Aldrich (St. Louis, MO). The MICs of meropenem (0.375 to 48 μg/ml), tobramycin (8 to 1,024 μg/ml), ceftazidime (8 to 1,024 μg/ml), and PAAG (8 to 1,024 μg/ml) were determined against each bacterial strain using the Clinical and Laboratory Standards Institute (CLSI)-approved microtiter serial dilution method (31). Serial 2-fold dilutions of each antibiotic and PAAG were made in MHB and aliquoted into 96-well flat bottom microtiter plates. Bacteria were grown overnight in MHB at 37°C and diluted to a 1 McFarland turbidity standard in MHB and added to each well. Untreated bacteria in MHB were used as controls. The plates were incubated at 37°C for 24 h. The MIC was confirmed by visual inspection showing inhibition of growth. The isolates were categorized as sensitive, intermediate, or resistant according to the CLSI guidelines (31).

### Effects of PAAG against preformed biofilm.

Biofilm formation was confirmed using a static tissue culture plate (TCP) method (44). The Bcc isolates were grown overnight in MHB and then adjusted to a 1 McFarland turbidity standard, which was further diluted 1:30 in MHB. The diluted culture was seeded into a 96-well microtiter plate. The Bcc biofilms were grown for 48 h at 37°C without shaking. The biofilms were gently washed twice with water. Then the biofilms were treated with PAAG (50 to 200 μg/ml) formulated in 1.38% glycerol, pH 7.4 (SYGN113), 3.2 μg/ml dornase alpha, or 7% hypertonic saline and incubated at room temperature for an hour. After an hour, the wells were rinsed twice with water and dried for 2 h at 37°C. The biofilms were stained with 1% crystal violet for 15 min. Stained biofilms were washed twice with water, and crystal violet was eluted in absolute methanol. After incubation for 5 min, the solubilized crystal violet was transferred into a fresh microtiter plate and the optical density (OD) was read at 590 nm. The average absorbance of biofilm-forming isolates was greater than the average absorbance of the negative control wells plus or minus 3 standard deviations and confirmed biofilm formation (44).

To test the minimum biofilm eradication concentration (MBEC) of PAAG against biofilm-forming Bcc isolates, bacteria were grown overnight in MHB and then adjusted to a 1 McFarland turbidity standard, which was further diluted 1:30 in MHB. The diluted bacteria were added to the trough, and the peg-lid was placed on top of the trough. The bacteria grew on the pegs for 48 h at 37°C on a rocking table set to between 3 and 5 rocks per minute with a 10° inclination (MBEC high-throughput [HTP] assay; Innovotech). After the 48-h incubation, the pegs were rinsed twice with water and placed in a 96-well flat bottom microtiter plate containing 50 to 200 μg/ml of PAAG formulated in 1.38% glycerol, pH 7.4 (SYGN113), and incubated at room temperature for an hour. Then the pegs were placed in a recovery plate containing neutralization broth and sonicated to dissociate the remaining biofilm from the pegs. To calculate the MBEC, biofilms were serial diluted spot plated onto MH agar plates and incubated for 24 h at 37°C. The initial bacterial inoculum and biofilm controls were also confirmed by serial dilution and spot plating.

### CLSM imaging of with LIVE/DEAD staining of PAAG-treated biofilms.

The bacteria were grown on 0.18-mm glass coverslips overnight in MHB and then adjusted to a 1 McFarland turbidity standard (41). The bacterial culture was further diluted 1:30 in MHB and seeded into each well of a 12-well tissue culture plate. The coverslips were gently placed into each well at a 90° angle relative to the bottom of the wells so that the meniscus of the medium was at the center of the coverslip. The bacteria grew on the coverslip for 48 h at 37°C. The nonadherent cells were removed following a gentle rinse with sterile water. The biofilms were treated with PAAG or PAAG-Cy5.5 conjugate for 1 h. The biofilms were rinsed twice and stained. The BacLight LIVE/DEAD bacterial viability kit (Molecular Probes, Eugene, OR) was used to stain the Bcc biofilms and prepared according to the manufacturer’s instructions. The coverslips were washed again to remove any excess stain and observed with a 63× lens objective by CSLM using a TCS SP5 microscope and software (Leica) with an excitation at 488 nm and emission detected using a dual-band emission filter (500 to 550 nm/598 to 660 nm).

### Effect of PAAG on optical thickness.

Light microscopy was used to measure the optical biofilm thickness of 48-h biofilms grown on coverslips. Optical thickness was translated into physical thickness based on biofilm refraction measurements. Biofilm refractive index was approximately equal the refractive index of water (32). Biofilm thickness, Lf, is proportional but not equal to the measured vertical displacement. The physical length is calculated using Lf ≈ kfyf, where yf is the optical thickness measured and kf is a proportionality constant function of the refractive indices of the biofilm and air. In view of the fact that the proportionality constant kf ≈ nf/na (refractive index of the biofilm [nf = 1.33 or approximately that of water] divided by the refractive index of air [na = 1.0]), the proportionality constant is approximately equal to 1.33. Therefore, the actual length (Lf ≈ kfyf) is measured by multiplying optical distance (yf) by the constant 1.33 (kf) (32). For every experiment, at least 25 depths of the biofilm were measured for each set of slides prepared. A total of three separate slides were used for every Bcc strain tested. The mean of all thicknesses measured from each slide was used to calculate the final thickness of the biofilm.
